# Perspectives on emerging challenges and training needs for the Laboratory Medicine specialist of the future

**DOI:** 10.1515/almed-2025-0146

**Published:** 2025-10-29

**Authors:** Giuseppe Lippi, Agostino Ognibene

**Affiliations:** Section of Clinical Biochemistry, University of Verona, Verona, Italy; Department of Laboratory and Trasfusional Medicine, AUSL Toscana Sudest, Arezzo, Italy

**Keywords:** Laboratory Medicine, specialists, future, competency, skill, expertise

## Abstract

Laboratory Medicine has undergone a radical transformation since its early 20th-century origins, evolving from rudimentary hospital laboratories into a technologically advanced discipline. Laboratory Medicine specialists now play multifaceted roles that extend beyond traditional analytical tasks to include clinical liaison, technological leadership, laboratory management and complex data interpretation within multidisciplinary healthcare teams. They are central to designing patient-centered diagnostic services, integrating automation and digital systems across increasingly networked laboratory infrastructures. Nevertheless, current challenges like enhancing test complexity, workforce shortage, budgetary constraints and integration of emerging digital technologies need continuous professional development and organizational adaptability to maintain quality and efficiency. The Laboratory Medicine specialist of the future will need to combine advanced technical expertise with transversal skills including leadership, communication, informatics and ethical governance. Proficiency in data analytics, network and system integration, and digital health platforms will enable Laboratory Medicine specialists to interpret complex biomedical data and optimize laboratory networks. Equally critical is commitment to sustainability and equity, encompassing green laboratory practices, scalable diagnostics and equitable access to services. Strategic workforce planning, advanced technical expertise, leadership, informatics, network optimization, sustainability-focused practices, educational innovations and structured mentorship programs are essential to cultivate these versatile professionals. This transformation establishes Laboratory Medicine not only as a technical cornerstone, but also as a strategic driver of patient-centered, equitable and sustainable healthcare.

## Historical evolution of Laboratory Medicine

The evolution of Laboratory Medicine has been driven by a dynamic interplay between technological innovations and growing clinical needs [1]. In the early 20th century, hospital laboratories were modestly outfitted; balances and light microscopes formed the core of diagnostic tools, serving largely localized needs with limited throughput and modest analytical and clinical performance [2]. The mid-20th century marked a pivotal expansion, with invention of the UV-visible spectrophotometer in 1940 by Arnold Beckman, especially the iconic Beckman DU model, which revolutionized the quantitative analysis of several analytes [3]. Concurrently, techniques like flame photometry and electrophoresis broadened test capabilities, enabling more refined assessments of several clinically important biomolecules [4].

By the late 20th century, this momentum continued with introduction of automation, quality control systems, and early Laboratory Information Systems (LISs) [5]. Such innovations have dramatically contributed to improving workflow efficiency, reducing errors and enabling a more standardized approach to data management, leading the way to the modern laboratory ecosystem. Almost simultaneously, the molecular diagnostics revolution began to reshape laboratory diagnostics. The late 1980s saw the emergence of the polymerase chain reaction (PCR), a disruptive innovation that enabled sensitive and specific detection of nucleic acids [6]. Variants such as digital PCR (dPCR) enabled absolute quantification and rare genetic variants detection over time [6]. The broader molecular field expanded rapidly, driven by advances like the Human Genome Project (HGP) and transitioned from laborious manual workflows to fully integrated, high-throughput systems [7].

Entering the 21st century, high-resolution mass spectrometry (MS), especially liquid chromatography coupled with tandem MS (LC-MS/MS), has emerged as a cornerstone of advanced diagnostics due to its unparalleled sensitivity and multiplexing capacity [8]. Routine application permeated many larger laboratories, where LC-MS/MS is now the gold standard approach for specific applications such as therapeutic drug monitoring (TDM), quantification of hormones and their metabolic products, as well as confirmation testing of immunoassays [8].

As laboratory complexity grew, so did the interest in integrated diagnostics [9], a paradigm that blends laboratory data with imaging and pathology insights through advanced IT infrastructures to enable a more accurate diagnosis and personalized treatments. The emergence of omics technologies (i.e., genomics, epigenomics, proteomics, metabolomics, and theragnostic) has then guided the need for high-throughput instrumentation, complex data analytics, and multidisciplinary collaboration, requiring novel cultural and organizational paradigm shifts in laboratory diagnostics [10], 11].

Approaching modern-day practice, the integration of Next-Generation Sequencing (NGS) and digital pathology platforms has become a hallmark of precision medicine, enabling molecular-level insights, tailored therapeutic strategies, and artificial intelligence (AI)-assisted image interpretation [12]. Automation in Laboratory Medicine has deeply revolutionized diagnostic practice since its widespread introduction at the end of last century [13]. Initially developed to address the growing test volumes of high-throughput clinical chemistry laboratories, early random-access analyzers and robotic sample handlers markedly reduced manual pipetting errors and turnaround times [13]. Laboratory automation systems can now integrate preanalytical, analytical, and postanalytical activities in fully connected workflows with features such as automated sorting, centrifugation, aliquoting, storage and retrieval of diagnostic samples [13], 14]. These systems not only have contributed to enhance efficiency, but have also significantly improved quality, traceability and reproducibility. Nowadays, automation is extending far beyond core clinical chemistry and hematology laboratories into molecular diagnostics, microbiology and pathology, increasingly incorporating digital systems for workflow optimization and predictive maintenance [15]. Far from displacing laboratory professionals, automation has redefined their roles, shifting emphasis from repetitive technical tasks to oversight, data interpretation and expert counseling.

This remarkable technological progression has been accompanied by a fundamental shift in the professional skill set required of Laboratory Medicine specialists [16]. There is now pressing demand for integrative competencies, combining multiple and complex technical expertise [17]. Simultaneously, contemporary thinking in value-based Laboratory Medicine (VBLM) emphasizes test appropriateness, clinical utility, sustainability, eco-friendly practices, and integration of advanced technologies, including AI, across all phases of the total testing process [18].

## Role and skills of today’s specialist

In contemporary healthcare, Laboratory Medicine specialists have a multifaceted role that extends far beyond traditional analytical tasks. They serve not only as diagnosticians, but also as leaders, quality stewards, and integral members of multidisciplinary clinical teams. Modern specialists are expected to synthesize complex diagnostic data to inform patient management, bridging the gap between laboratory test results and clinical decision-making [19].

Educational and professional standards have evolved to reflect this complexity, requiring proficiency across a vast array of specific domains like clinical chemistry, immunochemistry, hematology, hemostasis, microbiology, molecular diagnostics and, last but not least, clinical informatics [20]. Competence in all these areas is complemented by skills in laboratory and personnel management, process optimization, budgeting and quality assurance [21].

Operational responsibilities frequently encompass laboratory organization, method validation, workflow standardization, and regulatory compliance. Laboratory Medicine specialists are also crucial for facilitating clinical liaison activities, supporting evidence-based pathways that enhance appropriateness, diagnostic efficiency and patient outcomes [22], 23]. Laboratory Medicine specialists also play a pivotal role in technological innovation and digital transformation, overseeing the integration of automation platforms, point-of-care testing (POCT) devices and LIS with the Hospital Information Systems (HIS) [24]. The convergence of technical expertise, leadership capacity, and informatics acumen positions the modern Laboratory Medicine specialist as a critical architect of high-quality, patient-centered diagnostic services.

## Current challenges

The field of Laboratory Medicine is navigating an increasingly complex landscape, characterized by interrelated challenges that threaten both operational capacity and workforce sustainability (Table 1). Laboratory services are experiencing continuous growth in the quantity and quality of tests, driven by demographic trends such as population expansion, aging, rising prevalence of chronic diseases, increasing adoption of personalized medicine, as well as the growing demand for resilience in the face of natural disasters (e.g., earthquakes, pandemics, floods) and human-made crises (e.g., armed conflicts, industrial accidents, environmental degradation) [25]. These trends have placed unprecedented demands on laboratory infrastructure, straining limited human, technological and financial resources. Laboratories are simultaneously required to maintain rapid turnaround times, high analytical accuracy, and rigorous quality control standards, often under the scrutiny of resource-constrained healthcare systems.

**Table 1:** Current challenges in Laboratory Medicine.

Challenge	Description	Impact
Test volume and complexity	Aging populations, chronic diseases, personalized medicine increase demand	Strain on labs, need for rapid turnaround and accuracy
Workforce shortages and burnout	Aging workforce, pandemic impact, recruitment, and retention difficulties	Increased workload, quality risks
Economic and operational stress	Budget constraints, admin and managerial duties, undercompensation	Limits investment, adds stress and burnout
Emerging technologies integration	AI, ML, digital pathology require investment, technical skills, validation	Role changes, evolving training needs

AI, artificial intelligence; ML, machine learning.

At the same time, the technical complexity of laboratory assays is escalating [25]. Advanced multi-omic tests such as genomics, epigenomics, proteomics and metabolomics require sophisticated interpretation, highly specialized skill sets, and prolonged training periods to achieve full competency. These assays not only demand analytical accuracy, but also specific understanding of clinical contexts, bioinformatics and variant interpretation frameworks. The growing reliance on such complex diagnostics has intensified the need for continuous professional development, amplifying the risk of errors in laboratories with insufficient or insufficiently trained staff.

Workforce shortages in Laboratory Medicine are not a new phenomenon; they have been developing over several years due to a multitude of factors such aging workforce, limited recruitment, high attrition and comparatively lower compensation [26]. These longstanding shortages have been sharply amplified by the coronavirus disease 2019 (COVID-19) pandemic, which placed extraordinary stress on laboratory systems, accelerated retirements and exacerbated burnout among clinical laboratory professionals, as clearly emphasized by a survey endorsed by the European Federation of Clinical Chemistry and Laboratory Medicine (EFLM) [27]. The pandemic also highlighted vulnerabilities in global and regional workforce distribution, with rural and under-resourced laboratories experiencing more than others staffing deficiencies. Despite gradual recovery in some regions, workforce insufficiencies remain now evident, posing ongoing risks to laboratory activity, timely diagnostics and, ultimately, to patient care and safety [28]. Some laboratories are facing a dearth of qualified scientists, technologists and subspecialists [17], 28]. This shortage of personnel not only increases the relative workload per single individual, but also heightens the risk of burnout, impair job satisfaction and can eventually have a negative impact on analytical quality and laboratory throughput [29]. Many of these challenges also vary according to national healthcare contexts, reflecting the vast heterogeneity across Europe and abroad. Some countries have placed larger emphasis on laboratory consolidation and automation, whereas others often contend with more fragmented structures and more pronounced workforce shortages. These differences may influence both the severity of resource limitations and the approaches that are most effective for achieving sustainable laboratory organization.

Economic and operational pressures add another aspect of complexity. Laboratory services are frequently tasked with maximizing cost-efficiency while maintaining high-quality analytical performance [30]. Budgetary constraints limit the ability to expand infrastructure, invest in automation or implement comprehensive training programs. In addition to their technical and scientific duties, Laboratory Medicine specialists are increasingly tasked with extensive administrative and managerial responsibilities that are critical to operation, compliance and strategic planning of laboratory services, and which may then contribute to occupational stress [31]. These duties mainly encompass comprehensive activities within LIS/HIS, including test validation, result verification, and reporting. Laboratory Medicine specialists are also responsible for financial and resource management, including annual budget planning, monitoring of consumables and costs, and oversight of budgetary objectives set by the hospital administration. They play a pivotal role in procurement processes, from drafting technical specifications to managing tenders for laboratory instrumentation, while ensuring alignment with institutional policies and cost-efficiency goals. Maintaining documentation for certification and accreditation is another essential activity, involving development and upkeep of standard operating procedures (SOPs), tracking non-conformities, and implementing continuous quality improvement by means of internal and external quality assessment. Workforce planning responsibilities, such as organizing work schedules, shifts, leave and vacation planning, are integrated with broader organizational objectives, including participation in clinical pathways, preparation of technical reports, and provision of consultations.

Laboratory Medicine specialists are then accountable for regulatory and compliance activities, including continuing medical education (CME), legal safeguards and professional development beyond institutional CME requirements [32]. Additional tasks, such as managing electronic communications and responding to public inquiries, further underscore the multifaceted administrative load, demonstrating that modern Laboratory Medicine specialists must balance complex operational, regulatory and managerial duties alongside their scientific and clinical contributions. Overall, Laboratory Medicine is increasingly approaching the status of a “physically and mentally demanding” profession due to chronic workload overload and personnel shortages. Laboratory Medicine specialists face enhanced responsibilities with direct implications for clinical risk, coupled with the emotional stress of high-stakes decision-making. This burden is often exacerbated by limited recognition and insufficient gratification, both economic and professional, which can contribute reduced job satisfaction and difficulties in workforce retention [33].

Emerging technologies offer potential mitigation strategies, but inevitably introduce new challenges. Automation, AI and machine learning (ML) tools have begun to streamline workflows, improve data interpretation and enhance predictive analytics. Nevertheless, their integration needs substantial capital investment, technical expertise and ongoing validation to meet regulatory standards [34]. The adoption of digital pathology, robotic sample handling, and AI-driven decision support systems also requires the redefinition of professional roles, updated training programs, and development of ethical frameworks to extend governance over complex decision-making processes.

Despite their critical role in patient care, Laboratory Medicine specialists are frequently undercompensated compared to other healthcare disciplines due to several factors that have contributed to this wage disparity [17]. First, laboratory work is often less visible to patients and general public, reducing perceived value compared to frontline clinicians. Second, the traditional hospital and healthcare reimbursement models often prioritize billable patient encounters over laboratory services, limiting the financial recognition of laboratory contributions [35]. Third, historical underinvestment in laboratory infrastructure and personnel has created systemic salary stagnation. Fourth, Laboratory Medicine specialists have faced a tight labor market, in part because of limited advocacy and weak professional representation, which has left them with less leverage to negotiate better wages. Finally, gender and demographic factors may play an adjunctive contribution. Collectively, these factors contribute to persistent underpayment, which can exacerbate workforce shortages, decrease job satisfaction and hinder recruitment and retention of highly skilled laboratory personnel.

## Key skills of the future Laboratory Medicine specialist

The future of Laboratory Medicine requires a paradigm shift in the professional skill set, moving beyond traditional scientific and technical expertise to embrace a broader range of transversal, or non-technical, competencies (Table 2).

**Table 2:** Key skills of future Laboratory Medicine specialists.

Skill category	Specific skills and knowledge	Role implications
Advanced technical expertise	Automation, NGS, flow cytometry, digital pathology, MS, multi-omics, bioinformatics	Complex analysis and precision medicine support
Transversal/leadership skills	Communication, teamwork, leadership, management, ethics	Multidisciplinary collaboration and strategic laboratory management
Informatics and data analytics	Machine learning, predictive modeling, data visualization	Interpretation of big data for clinical insights
Network and system integration	Lab network management, LIS/HIS interoperability, quality assurance	Ensuring standardized, efficient, and patient-centered services
Sustainability and equity focus	Green chemistry, resource efficiency, global health equity	Environmentally responsible and equitable healthcare delivery

NGS, next-generation sequencing; MS, mass-spectrometry; LIS, Laboratory Iinformation System; HIS, Hospital Information System.

Laboratory medicine specialists will increasingly require a broader competency that integrates deep technical-scientific mastery with essential transversal skills, positioning them as technical virtuosos and collaborative leaders. This evolution is directly supported and expanded upon by the EFLM syllabus [36], 37], which provides a comprehensive framework for postgraduate education and training. By integrating the core principles of the syllabus with the emerging demands of a data-driven healthcare landscape, we can define a new profile of Laboratory Medicine specialist, i.e., one capable of performing complex analyses, interpreting high-dimensional data, managing a multifaceted laboratory environment and contributing strategically to healthcare innovation.

On the technical side, future expertise will include advanced methods and cutting-edge diagnostic tools such as NGS, single-cell analysis, flow cytometry, digital pathology, MS and multi-omic profiling. The EFLM syllabus version 5 emphasizes “new analytical techniques and use of statistics” and recognizes the growing importance of omics technologies within its “basic knowledge requirements” [37]. Specialists will also need to analyze and interpret complex clinical datasets using computational tools such as predictive modeling and AI, enabling the extraction of actionable insights to support precision medicine initiatives. Oversight of POCT will represent a critical responsibility, requiring stringent quality management, clinical integration across evenly distributed health care settings, and full adherence to regulatory standards. Proficiency with laboratory automation, robotics, and laboratory information management systems will remain essential for managing increasing test volumes, workflow complexity and operational efficiency.

Equally critical are transversal competencies that enable effective participation within multidisciplinary healthcare teams. The EFLM syllabus [37] explicitly addresses leadership, communication, teamwork, and management skills, including research, development, and audit activities. Trainees are expected to gain experience communicating with and collaborating alongside clinical and non-clinical colleagues, participating in team, management and leadership forums. Specialists also serve as stewards of sensitive patient data, requiring integrity, judgment, and compliance with governance frameworks. Advanced informatics capabilities, including database management, bioinformatics and data visualization, position Laboratory Medicine specialists as central “(big) data interpreters”, who are capable to translate complex technical test results into meaningful clinical information [38].

An important advancement in Laboratory Medicine involves the strategic consolidation and streamlining of laboratory networks to enhance operational efficiency, process standardization, and long-term sustainability, introducing the new concept of “global-of-care testing” (GOCT) [39]. This model envisions a globally interconnected diagnostic infrastructure that combines regional flexibility, robust governance, and sustained investment in both technology and human resources. The former movement toward larger, centralized laboratories (Hubs) serving multiple smaller peripheral laboratories (Spokes) has been motivated by the dual objectives of cost containment and optimized workflow efficiency [40], 41]. Effective planning of laboratory networks, however, must also consider geographic distribution and population density to guarantee equitable access to services. Integrated systems should be based on automation that can connect diverse analytical platforms, streamlining workflows, enabling real-time clinical decision-making, facilitating remote collaboration and maintaining rigorous quality standards. A decentralized but interconnected model allows peripheral laboratories to play an active role in patient care through harmonized protocols, integration of telemedicine, and shared data systems, ultimately reducing turnaround times, improving responsiveness and supporting patient-centered care. Equally critical is standardization of analytical methods and implementation of a unified quality management framework, which are essential to ensuring accuracy, reproducibility and comparability of test results both within and beyond the laboratory network [42]. The integration of information systems, especially interoperable LIS and HIS, is essential to ensure seamless communication, data exchange and system compatibility across facilities. Optimized logistics and sample transport must maintain accurate transport conditions, ensure traceability and establish clear criteria for sample acceptability, safeguarding sample integrity and diagnostic reliability.

**Figure 1:**
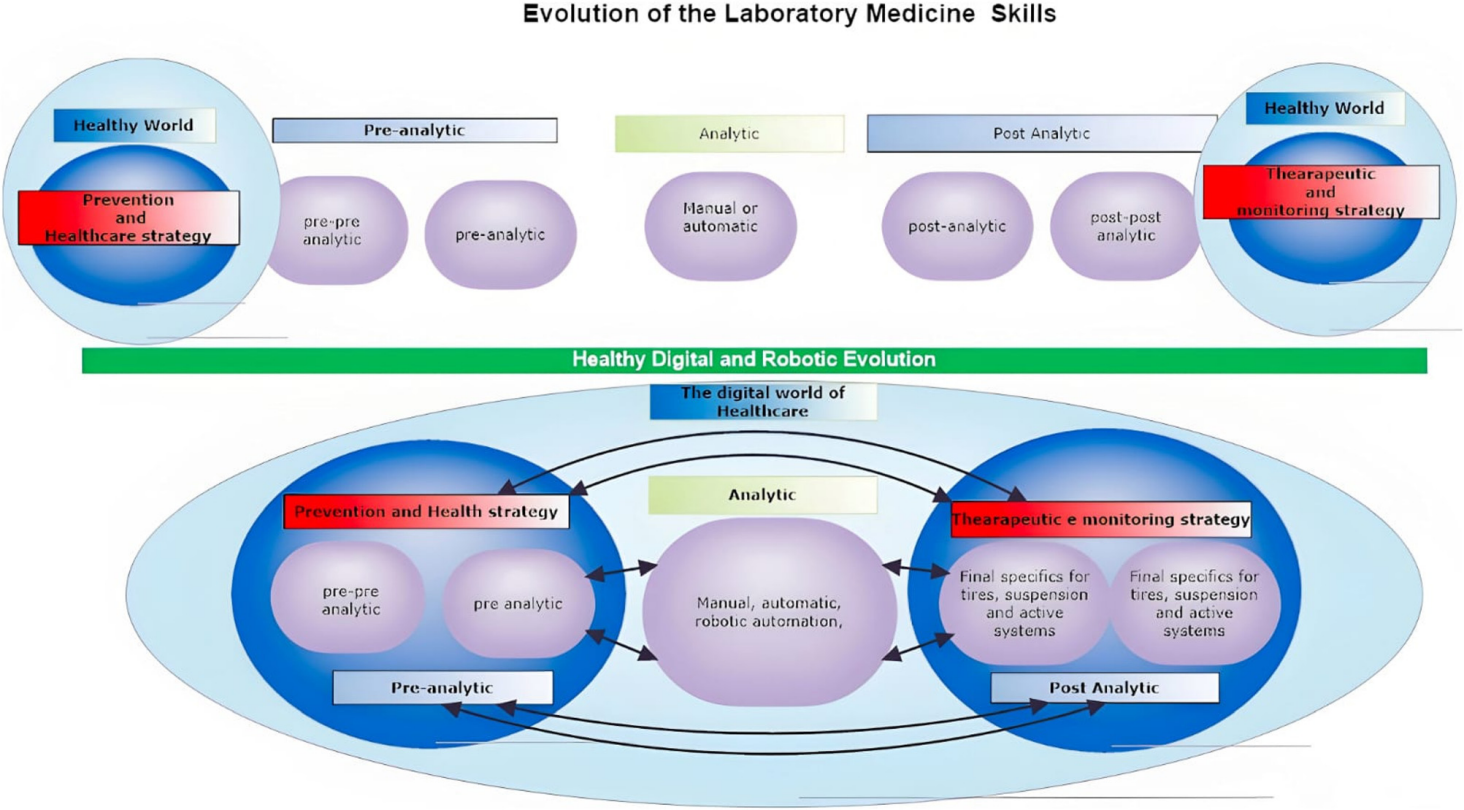
Increasing integration of system connectivity, which have enabled enhanced connectivity and development of healthcare environments characterized by integrated communication.

Beyond the substantial technological advancements in laboratory analytics previously outlined, the most significant transformation in Laboratory Medicine is the growing integration of system connectivity [43]. This enhanced interoperability has enabled the creation of healthcare environments defined by seamless communication. Supported by consistent and reliable networks, such connectivity fosters new forms of collaboration and information exchange, equipping professionals with a comprehensive and up-to-date view of patient information. Advancements within this ‘Cloud’ (Figure 1) require competencies that integrate epidemiological expertise, evidence-based selection of screening tests, and proficient and sustainable management of the analytical phase. The ultimate outcome is a personalized diagnostic pathway, delivered through an innovative report that synthesizes the clinical event with the patient’s medical history, providing tailored guidance for both acute and chronic conditions.

Within this context, laboratory professionals are expected to possess not only advanced clinical expertise that surpasses traditional isolated practices, but also proficiency in leveraging interactive communication tools, interpretative algorithms and AI-driven solutions to support their roles. It is crucial that these core competencies complement, rather than detract from, ongoing engagement with continual technological advancements that shape both the technical and organizational dimensions of laboratory processes. Moreover, enhanced connectivity facilitates the involvement of new employees and students in on-the-job training, allowing them to engage directly with emerging innovations and benefit from the expertise of seasoned practitioners.

**Figure 2:**
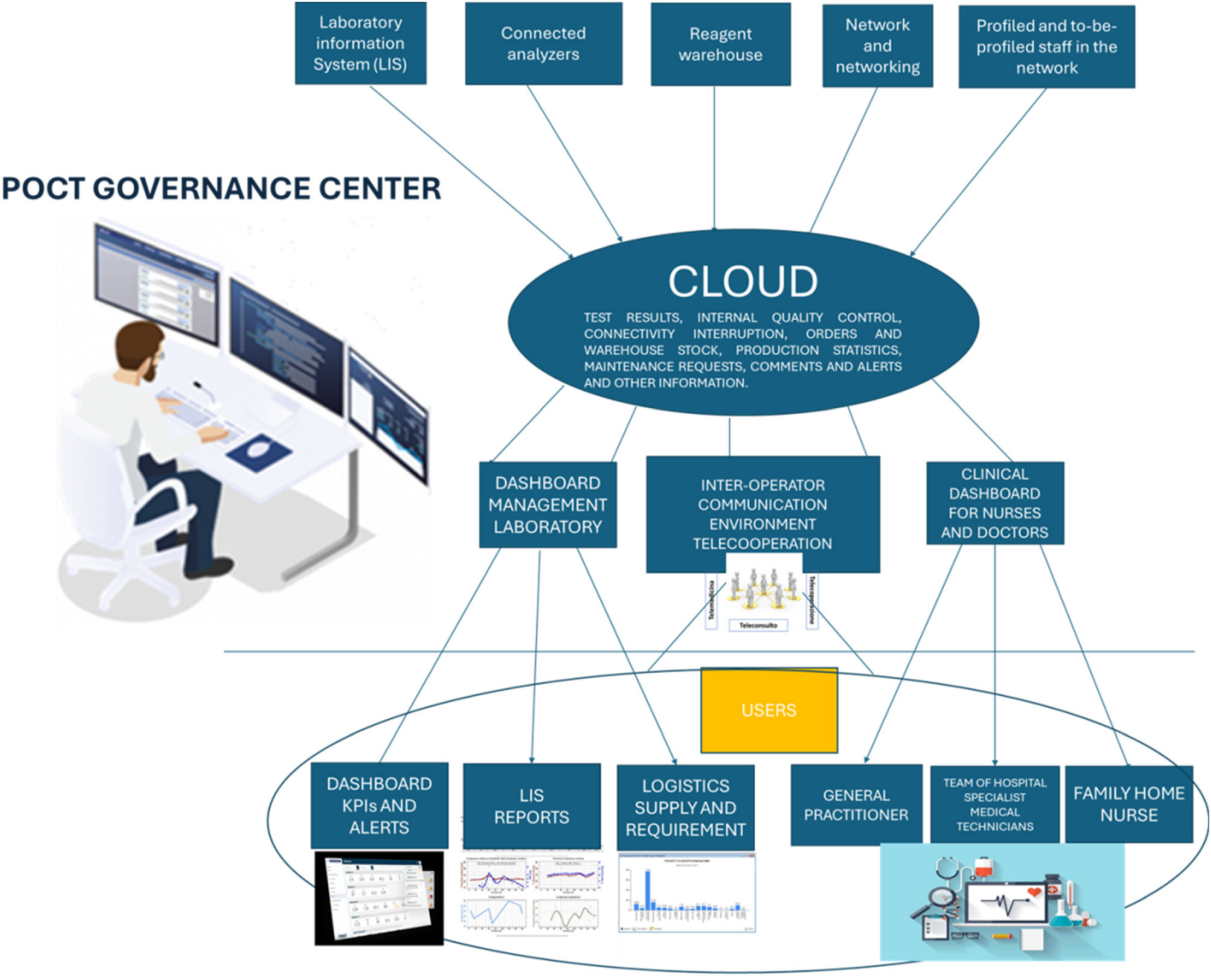
Operation of governance platforms for point of care testing (POCT) instrumentation networks used to monitor patients with chronic diseases at home.

As previously described, a complex activity structured under these assumptions is the operation of governance platforms for POCT instrumentation networks, which are typically used for home monitoring of patients with chronic diseases (Figure 2) [44]. Network users engage in preparation and training through a clinical and diagnostic virtual demohouse, which provides updates on the latest patient-monitoring technologies. Practitioners will access dashboards displaying real-time data and reports generated during monitoring sessions, including continuous records of organ function and laboratory diagnostic results.

Bringing together technical expertise, cross-cutting skills and efficient network organization, Laboratory Medicine specialists are evolving into a versatile and strategic partner in healthcare. They will not only perform sophisticated tests, but also guide the implementation of new diagnostic technologies, interpret and communicate results within multidisciplinary teams, oversee centralized laboratory networks and ensure that operations comply with ethical, regulatory, and quality standards. In this holistic vision, Laboratory Medicine specialists will actively contribute to the evolution of healthcare, supporting patient-centered, data-driven medicine while ensuring scalable, standardized and economically sustainable laboratory networks that will also need to shift towards green practices, resource efficiency and reduced waste operations. Consequently, workforce development programs should prioritize advanced scientific training, leadership development, health informatics, ethical proficiency, and strategic management skills to equip specialists for a technologically advanced and operationally complex future. These programs should also foster interdisciplinary integration, transcending traditional silos across different diagnostic disciplines.

Educating the future Laboratory Medicine specialists demands innovative curricular designs that transcend traditional didactic approaches. Blended learning models that combine interactive online content with hands-on laboratory training help students to remain engaged and build practical skills. Simulation-based teaching provides a safe way to practice handling laboratory crises and interpreting test results. Interdisciplinary specialization or master PhD programs strengthen research skills and encourage collaborative problem-solving. New tools such as massive open online courses (MOOCs) and virtual labs make education more accessible worldwide and support ongoing professional development. Mentorship and residency programs remain essential for guiding individual growth and preparing professionals for work in clinical settings [45]. International organizations such as the EFLM and the International Federation of Clinical Chemistry (IFCC) play a key role in aligning curricula and promoting the translation of research into practice. Finally, embracing lifelong learning is crucial to keep pace with rapid technological change and ensure the laboratory workforce continues to deliver excellence.

As previously emphasized, integrating laboratory systems with digital health platforms expands the role of Laboratory Medicine specialists in telemedicine and home-based diagnostics, enhancing patient access to healthcare. However, robust cybersecurity measures and comprehensive data protection policies will become increasingly pivotal to safeguard patient information, ensure regulatory compliance and manage the growing volume of electronic health records and big data [46].

Beyond technical and educational considerations, additional domains will substantially reshape the future trajectory of Laboratory Medicine. Although this opinion paper often refers to Laboratory Medicine as a whole, it is important to recognize its main constituent specialties, i.e., Clinical Chemistry, Hematology, Immunology, Genetics (Molecular Biology), and Microbiology. Each of these requires distinct expertise, and in daily practice the greatest contribution to clinical interpretation often comes from subspecialization, sometimes even within these broader fields. Environmental sustainability is also paramount, given the significant resource consumption and waste generation inherent in laboratory processes [47]. Adoption of green chemistry principles, energy-efficient technologies and reduction programs for single-use plastics and reagents are becoming increasingly necessary [48], 49]. Finally, promoting global health equity is an ethical priority. Training programs should include scalable, low-cost diagnostic approaches that can be adapted to resource-limited settings, helping improve health outcomes. At the same time, ensuring fair compensation and professional recognition is essential to reduce workforce attrition and burnout. Laboratory Medicine specialists will also need strong communication skills to engage patients effectively, providing clear and empathetic explanations of complex test results.

## Conclusions

Since its origins, in the early 20th century, Laboratory Medicine has undergone a profound transformation, evolving from rudimentary hospital laboratories into a technologically sophisticated discipline. It now stands at a pivotal crossroads, with ongoing advancements shaping its future trajectory. Addressing the challenges outlined in the previous sections of this opinion paper demands multifaceted strategies, including strategic workforce planning, expanded educational pathways, targeted recruitment and retention initiatives, investment in automation and decision-support technologies, along with policy frameworks that promote both operational efficiency and sustainability. Failing to act could compromise laboratory capacity and efficiency, with significant consequences for patient care, clinical decision-making, and advancement of personalized medicine.

To effectively face these emerging challenges and seize opportunities, coordinated strategic initiatives are essential. Key steps include creating shared national and international training standards through organizations like the EFLM and IFCC, encouraging collaboration between universities, public and private laboratories, diagnostic industry and professional societies, as well as establishing mentorship and career development programs to attract and keep young talents.

Investment in research focused on Laboratory Medicine innovations will foster technological adoption and support evidence-based practice. Comprehensive workforce planning must also consider current and future demographic changes and supply-demand imbalances, while considering global challenges such as dwindling natural resources (e.g., petroleum) and ongoing conflicts. Educational programs should integrate sustainability and digital health priorities to prepare a resilient and adaptable laboratory workforce. Continuous program evaluation and iterative curriculum updates will ensure responsiveness to evolving technological and societal needs. Ultimately, the transformation of Laboratory Medicine specialists into versatile, analytically skilled, and communicative professionals is critical to safeguarding healthcare quality and patient outcomes. Prioritizing this transformation is essential for the sustainability of health care systems worldwide [50].

In conclusion, Laboratory Medicine is evolving beyond traditional analytical roles toward comprehensive responsibilities that integrate governance of interconnected diagnostic infrastructures, data interpretation, interdisciplinary collaboration, patient-centered care and VBLM. Future specialists in Laboratory Medicine will not only ensure analytical accuracy but also interpret complex biomedical data, helping the advancement of precision medicine. Emerging therapies in oncology and other medical fields, such as novel immunotherapies, also highlight the increasingly central role that Laboratory Medicine will play in monitoring treatment response, guiding clinical decisions, and supporting precision medicine in the near future. Finally, education of the future generation of Laboratory Medicine specialists should include scientific expertise, digital literacy, ethical responsibility and adaptability. Meeting this mandate requires sustained investment in training, ongoing professional development and robust support frameworks. Such commitments will ensure that Laboratory Medicine continues to play a vital role in health system innovation, sustainability, and improved patient outcomes.
